# Standardized natural product cannabis in pain management and observations at a Canadian compassion society: a case report

**DOI:** 10.1186/1757-1626-2-7487

**Published:** 2009-05-18

**Authors:** A Paul Hornby, Manju Sharma, Bree Stegman

**Affiliations:** 1 Department of Medial Cannabis Research, The Green Cross Society of BC2127 Kingsway, Vancouver, B.C., V5N 2T4Canada; 2 Department of Pathology and Laboratory Medicine, Heather Pavilion, Vancouver General HospitalVancouver, B.C.Canada; 3 Canadian Registered Nurses Association of B.C., Vancouver Coastal Health Authority2755 Arbutus Street, Vancouver, B.C.Canada

## Abstract

An adult Caucasian male with excruciating pains following multiple traumas was monitored, daily, over one year while managing chronic pain by self-administering quantifiable amounts of natural cannabis. Tetrahydrocannabinol, Cannabidiol, and Cannabinol were all measured in tinctures, capsules, smoke-able product plus some baked goods, prior to their administration. By allowing standardization, the subject was able to develop a daily regimen of pain management that was resistant to a battery of most patent analgesics.

## Introduction

The chronic pain resulting from a severe on-the-job injury is a frustrating experience both for the patient as well as the treating physician and leads to chronic dependence on opiate painkillers and anti-depressants. However the relief of pain may be less desired if quality of life of such individuals is poor. The physician and the patients are left with no option but to resort to alternative modes of therapy. Cannabis has been documented to be one of such measures, especially in advanced cases of cancer [1]. It holds an enormous potential as medicines derived from cannabis plant exhibit a phenomenon termed strain specific symptom relief. It has been documented to be of proven value in arthritis and multiple sclerosis; however no controlled clinical trial for its use in chronic intolerable pain is reported. Hence, this case report. The person involved in this study is a member of the Green Cross Society of British Columbia, which has Federal tax number to distribute cannabis for medical purposes. The Society provides natural product (cannabis, herbal medicine) to its qualified members.

## Case presentation

The volunteer, a 33 year old Caucasian male, volunteer was selected from the membership based on his record keeping ability, the severity of his injury, plus his daily presence at the Society, allowing continuous monitoring. The subject kept detailed notes of his condition, including pain charts, medications and dietary habits, allowing comparison by study observers. The individual's note taking allowed an in depth review of his condition. The case described here is strikingly similar to four others of its type, run over the same year, with comparable observations and outcome.

Three years ago the subject (then, 33 years of age) was in good health and employed as a glacier, when he fell 28 feet onto concrete. As a result of this fall he suffered multiple injuries including C6 vertebral, skull, left olecranon, left hip, ankle and trochanteric fractures with multiple disc herniations. He underwent many surgical procedures for the management of these injuries and continued with pain of levels are 5/10 on a constant basis.

Cervical fracture impaired his ability of upper body to 75% and he was unable to read or work on computer. He had tension headache and tinnitus due to fractured skull. Shattered left elbow caused muscle spasms and pain at times. Left wrist pain was constant at level 8/10. It was burning at times, ice cold and had hammer like effect. Left hand was hard to use. He had back spasms 6/8 on both sides due to spine injury. He also had moderate degree of pain in left hip and ankle joints. Subsequent treatment resulted in, two years of physiotherapy, plus a long list of medications including: Arthrotec, Flexeril, ketorolac, Tylenol 3 with codeine, Naprosyn, Percocet, gabapentin, Marinol, Lyrica, Supradol, oxycodin and Oxycontin for pain. Doxepin, Imovane, Cipralex, trazadone, Elavil, Effexor XR for depression and HCTZ, Lipitor and ranitidine for a secondary hypercholesterolemia, the subject was unable to achieve satisfactory pain relief using these medications. And, he complained that the medications turned him into “a living dead person”, unable to work, go to school and carry out normal daily functions.

### HPLC

Chromatography, using a Hewlett-Packard (Agilent) 1090 Series II binary pumping system with a 79883a diode array detector, with primary absorbance at 219 nm, was conducted. Mobile phase at 1 ml/min was isocratic with 14% aqueous (1:25:974 phosphoric acid: acetonitrile: distilled water) and 86% organic phase (acetonitrile). The column was a Zorbax C18 reverse phase 4.6 mm × 25 cm. Samples were prepared by the method modified from Health Canada for the preparation of hemp samples for HPLC analysis [2]. Calibration curves were run for a number of commercial standards (Sigma) and averages made. 0.1 gram of dry (dryness determination appendix 1) cannabis was suspended in 10 ml THF and sonicated for 3 minutes then passed through a 0.45 micron nylon syringe filter into a 1 ml HPLC sample vial. Under these conditions suitable chromatography is achieved for the three most abundant cannabinoids present in the samples provided to the Society's members.

The total mean concentration of cannabinoids for 30 random samples taken at the Society was; CBD plus CBD-A (5.6 ± 3.1), CBN plus CBN-A (5.1 ± 3.2) & THC plus THC-A (172 ± 26). Review of the literature of cannabinoid concentrations found throughout the world shows dramatic variation, not only of THC but the other cannabinoids and their ratios [3]. The cannabis supplied by the Society's contracted growers is optimized for THC concentration through genetic selection of specific strains, growing conditions and fertilizers. Organic growing conditions are a priority. The high THC levels are preferred by the Society's membership for pain and tremor relief.

From January of 2007 to April 2008 we monitored the cannabis use of the participant case. We accumulated data on the amount of smoke-able, encapsulated, edible and tincture preparations consumed by the member, on a daily basis. His prescription record, physician's notes, urine (drug) tests, plus daily interviews were maintained and examined. Daily cannabis use totaling 10 g of natural product cannabis, translating to an average of 420-500 mg of THC, 40-80 mg of CBD and 20-60 mg CBN, was required to achieve a sufficient degree of pain management. Significant reductions in daily pain scores as well as improved sleep, muscle spasm and general quality of life were achieved. Although, by no means pain free, the patient is now able to do some part time volunteer work, go to the gym, and lead what resembles a normal life. He consumes 10-15 g of cannabis per day. He also finds benefit in a number of supplements: for chronic pain and depression, including, GABA (500 mg), L- Tyrosine (500 mg), L-Tryptophan (550 mg), DL- Phenylalanine (500 mg) and S-adenosyl methionine (liquid) 40 drops a day. For the breakthrough pain he used cannabis tincture at 10 mg THC/drop; 2 mg CBD/drop: 15-25 drops (as needed), which relieved intense pain, in a couple of seconds. He also used Volcano (vaporizer), 2-4 g a day. Recent medical examination showed all liver functions to be normal, including clearance of the hypercholesterolemia.

## Discussion

The analgesic properties of cannabis are becoming well established in the literature [4]. However, it remains controversial as a medicine and even more so as a plant. The purpose of this case study was to observe the efficacy and usefulness of the standardized whole plant cannabis medicine. Indeed, the complexities of elucidating the efficacy of such preparations is a difficult task, yet the benefits of the natural product far outweigh the contrary in consideration of toxicity, efficacy and side-effects [5]. With regard to the latter, more frequently unwanted side effects from cannabis result from overdose than any other parameter. And, most frequently, this overdose results from oral ingestion of un-standardized baked goods (i.e. brownies). Overdose results in confusion, paranoia and fear that subsides after four to six hours, often into sleep. In no case, has it been observed to cause permanent physical or mental damage [6], but can often leave the individual with extreme caution to repeating the event. The second most frequently observed un-wanted side effects arise from incorrect strain selection for the symptom. For example, a person seeking pain relief and also suffering from anxiety, chooses a strain containing high concentrations of CBN, with little comparative CBD and low THC, may experience increased anxiety, with little or no pain relief. Another important observation is that there is a genealogical factor in tolerance experienced by individuals of different ethnic backgrounds. Persons of Celtic descent (Scottish, Irish or Welsh) appear to be 3 to 5 times more tolerant to cannabis than persons of middle European or African descent. The person described in this study had a Scottish mother, which may explain the high THC levels required by him, but not by persons in similar studies but of different ethnic background.

## Conclusions

The case reported here represents one of many observed at the Green Cross Society. With 70% of the members treating chronic pain the same phenomenon is observed over and over that people achieve a significant degree of pain management using standardized natural product cannabis. Often a better quality of life is attained with cannabis use only, or in conjunction with reduced opiate consumption. The subject in this study is nearly one year using only natural product cannabis plus supplements for his severe pain. He recently went through yet another two surgeries to back and hand using only cannabis for post-operative pain.

The roughly 4000 members of the Green Cross Society find similar benefit from standardized natural product cannabis medicine. To follow, will be publication of the Society's demographic data regarding use for various conditions such as arthritis, fybromyalgia, HIV/AIDS, and chronic pain, to name a few. A breakdown of the illnesses, what strains (cannabinoid profiles) is most effective, and at what dosages will be published at a later time.

## List of abbreviations

AIDS: Acquired immunodeficiency syndrome
CBD-A: Cannabidiolic acid
CBD: Cannabidiol
CBN-A: Cannabinolic Acid
CBN: Cannabinol
GABA: Gamma aminobutyric acid
HIV: Human immunodeficiency virus
HPLC: High Performance Liquid Chromatography
THC-A: Tetrahydrocannabinolic Acid
THC: Delta-9 tetrahydrocannabinol
THF: Tetrahydrofuran

## References

[bib-001] Mechoulam R, Peters M, Murillo-Rodriguez E, Hanus LO (2007). Cannabidiol - recent advances. Chemistry & Biodiversity.

[bib-003] Clarke RC (1981). Marijuana Botany.

[bib-004] Grotenherman F, Russo E (2002). Cannabis and Cannabinoids.

[bib-005] Cannabis (Drug). http://en.wikipedia.org/wiki/Marijuana.

[bib-006] Guy GW, Whittle BA, Robson PJ (2004). The Medicinal Uses of Cannabis and Cannabinoids.

